# Impact of SiO_2_ and TiO_2_ Nanoparticles on the Elasticity and Aging Resistance of Polyvinyl Acetate (PVAc) Adhesive

**DOI:** 10.3390/ma17235957

**Published:** 2024-12-05

**Authors:** Gorana Petković, Suzana Pasanec Preprotić, Marina Vukoje, Ivana Bolanča Mirković

**Affiliations:** University of Zagreb Faculty of Graphic Arts, Getaldićeva 2, 10000 Zagreb, Croatia; suzana.pasanec.preprotic@grf.unizg.hr (S.P.P.); ivana.bolanca.mirkovic@grf.unizg.hr (I.B.M.)

**Keywords:** polyvinyl acetate adhesive, nanoparticles, nano-modified adhesive, elasticity, aging resistance, UV radiation, adhesive bonding

## Abstract

Adhesive modification with nanoparticles affects multiple adhesives properties, making it essential to evaluate and compare changes across all key characteristics—existing positive and limiting properties. This study investigates the impact of silica (SiO_2_) and titanium dioxide (TiO_2_) nanoparticles on the elasticity and aging resistance of PVAc adhesive. Tensile properties were determined according to ISO 527-3:2018, with Young’s moduli of elasticity $\left(Ε\right)$, and stress–strain curves for neat PVAc, nano-SiO_2_ PVAc, and nano-TiO_2_ PVAc adhesive. Material toughness ($U_{T}$), failure stresses $\left(\sigma_{f}\right)$, and failure strains $\left(\varepsilon_{f}\right)$ were also calculated. After UV exposure (0, 48, and 96 h), according to ISO 9142:2003, samples were characterized by Fourier-transform infrared spectroscopy (FTIR). Analysis of variance (ANOVA) was performed to determine if there is a statistically significant difference in material toughness between neat PVAc and nano-modified PVAc adhesives, as well as changes in FTIR spectra of paper–adhesive samples before and after UV exposure. The Bonferroni post hoc test was used to identify specific group differences. The results showed that SiO_2_ nanoparticles improved PVAc elasticity by 9.15%, while TiO_2_ nanoparticles reduced elasticity by 44.47%. FTIR analysis revealed similar behavior in both nano-modified and neat PVAc adhesives after UV exposure, indicating that aging resistance was preserved with the addition of SiO_2_ or TiO_2_.

## 1. Introduction

Polyvinyl acetate (PVAc) adhesives are widely used in the graphic industry for bookbinding and packaging applications, where different materials such as paper, board, leather, and cloth can be used in adhesive joints. In the graphic industry, adhesive binding is growing in popularity due to advancements in adhesive binding technology, shorter delivery time, and the introduction of innovative materials. In order to overcome the main possible disadvantages of adhesives—such as weak mechanical strength and limited elasticity, low resistance to temperature and humidity changes, slow curing time and limited durability, low adhesion between adhesive and challenging substrates, and environmental and health unacceptability—in the last few years, there have been numerous studies dealing with the modification of adhesives with nanoparticles [1,2,3,4,5,6,7,8,9,10].

In graphic post-printing production, particularly bookbinding, three main types of adhesives are used to achieve an adhesive bond: the previously mentioned water-based PVAc adhesives, hot melt ethylene vinyl acetate (EVA) adhesives, and reactive hot melt polyurethane (PUR) adhesives [11]. The choice of adhesive type largely depends on the properties of the adherents, available technology, the binding process itself, and the production volume [12]. Polyvinyl acetate (PVAc) adhesives are used in handmade and short-run production, reactive hot melt polyurethane adhesives in long-run automatic line production, while ethylene vinyl acetate adhesives are characteristic for automatic middle-run production. PVAc adhesives are water-based adhesives suitable for standard post-printing applications that do not require high moisture or temperature resistance. They dry by evaporation of water and offer moderate bonding strength and elasticity. They are affordable and non-toxic. EVA hot melt adhesives are ideal for high-speed processes due to their fast setting time and resistance to aging and moisture. They can provide strong bonds, but conversely, they can become brittle at lower temperatures and with non-porous materials. Additionally, they are not as environmentally friendly as PVAc adhesives. PUR adhesives offer the highest bonding strength and durability, with excellent resistance to temperature and moisture. They are used in high-end graphic applications, are more expensive than PVAc and EVA adhesives, and require special application equipment for the curing process [13]. PVAc adhesives are becoming more popular, due to the development and growth in the number of audiobooks and e-books, i.e., the continuous growth of short-run book production. In addition, current trends for adhesive technology development are focused on replacing solvent-based adhesives with water-based adhesives while achieving equal or better adhesive bond quality [14,15]. Most of the existing research studies related to the modification of PVAc adhesives with nanoparticles have been focused on the addition of silica (SiO_2_), nanoclay (NC), cellulose nanofibrils (CNFs), and titanium dioxide (TiO_2_) nanoparticles [1,2,3,4,5,6,7,8,9,10]. A review of the literature shows that achieving better adhesive properties of PVAc and increasing its resistance to temperature changes can be accomplished by adding SiO_2_ and TiO_2_ nanoparticles [2,8], as well as NC and CNFs [1,5,9]. The use of SiO_2_ and TiO_2_ nanoparticles as stabilizers can increase the water resistance of PVAc and improve its mechanical and adhesive properties under conditions of elevated humidity and temperature [3,10]. Due to their excellent properties and affordable price, SiO_2_ nanoparticles are most used when modifying nanocomposites of polymer matrices (PMCs), such as adhesives, polymer films, and coatings. Generally, SiO_2_ nanoparticles are used to modify and improve the rheological properties of liquids, adhesives, and elastomers [6]. TiO_2_ nanoparticles have been less frequently used in research involving the modification of PMCs, and the mechanical strength results of such materials are often inferior to those of nanocomposites with SiO_2_ nanoparticles. However, the addition of TiO_2_ nanoparticles can reduce material degradation under UV radiation, ensure color stability, extend product lifespan, and shorten drying time [6,16]. The most significant property of TiO_2_ nanoparticles is still their well-known pigmenting ability, which is used in numerous polymer industries to enhance whiteness, brightness, and reduce material transparency [17]. Most of the literature dealing with PVAc adhesives is related to wood joints and the use of different natural or chemical modifiers to strengthen the performance of a PVA adhesive [18,19,20,21,22]. For the PVAc–wood adhesive joints, modifications are conducted to perform effectively under demanding conditions, such as low-temperature environments. Therefore, a key focus is on optimization of the modification process to make it more efficient and cost-effective while maintaining or improving performance [18]. The lack of research in the field of PVAc adhesive–paper systems emphasizes the problem in this area of interest, given the increasing trend of the graphic and packaging industries to replace polymeric materials with paper-based materials in order to improve the environmental sustainability of the aforementioned industrial sectors, where PVAc is one of the most common adhesives used [23].

Unfortunately, during adhesive modification, it is not possible to selectively change only one desired property, such as the strength of adhesive joint; thus, it is necessary to test, observe, and compare changes in all key properties. The modification of PVAc adhesive with nanoparticles is only successful if the adhesive retains its existing positive properties, such as satisfactory resistance to aging, invisibility of the dry adhesive film, and elasticity [12,24,25,26,27,28], with a positive effect of nanoparticles on its limiting properties: end-product strength, long drying time, and low resistance to temperature and humidity changes [12,27,28,29].

**Figure 1:**
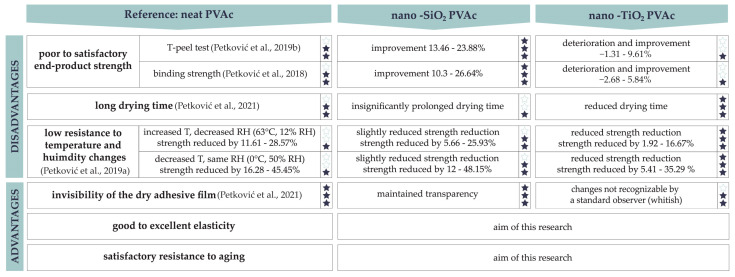
Changes in the PVAc adhesive’s properties after adding SiO_2_ or TiO_2_ nanoparticles based on existing research studies [30,31,32,33] (adhesives ranked according to the number of stars, 1–3).

Previous research has been focused on the investigation and provision of the positive impact of silica (SiO_2_) and titanium dioxide (TiO_2_) nanoparticles on the main disadvantages of PVAc adhesive. It has been shown that by adding SiO_2_ or TiO_2_ nanoparticles, besides improving thermal stability and durability [30], a considerable increase in bonding strength can be achieved [31,32]. Also, with the addition of SiO_2_ or TiO_2_ nanoparticles, it is possible to reduce the drying time and maintain the invisibility of the dry adhesive film [33]. The end-use strength test (T-peel) of adhesive joints indicated that nanoparticles could improve the bonding strength, especially SiO_2_, up to 23.88% [31]. Nanoparticles also improved the binding performance of PVAc adhesive, which was reflected in the binding strength and book-opening behavior. By adding SiO_2_ nanoparticles, the binding strength increased by up to 26.64% [32]. All the changes in the PVAc adhesive’s key properties investigated up to now, after modifications with SiO_2_ and TiO_2_ nanoparticles, are illustrated in Figure 1. To enhance clarity regarding the impact of the mentioned nanoparticles on the results of our previous research [30,31,32,33], adhesives were rated from one to three stars based on each property outlined in Figure 1.

In this study, PVAc adhesive was modified by adding 1% silica (SiO_2_) or 1% titanium dioxide (TiO_2_) nanoparticles in order to investigate the influence of this modification on PVAc elasticity and PVAc aging resistance. The optimum nanoparticle concentration in PVAc adhesive was defined in our previous research as 1% [31].

**Figure 2:**
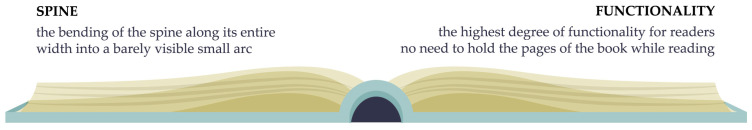
Book flat opening behavior.

The elasticity of PVAc adhesive stands out as one of PVAc’s main advantages. Usually, it directly affects not only the bonding strength but also the visual appearance and user experience of the end-product. For example, if the adhesive is elastic enough, it will be possible to create a high-quality book with exceptional properties: optimal binding strength and flat opening behavior (Figure 2). Unfortunately, the increase in elasticity of PVAc adhesive is most often associated with a decrease in cohesive strength and, thus, the overall strength of the end-product.

During storage and use, end-products are exposed to frequent temperature changes and fluctuations in humidity [33]. The quality of the finished products depends on their resistance to these changes and aging. The aging of paper in books is the result of complex chemical and physical processes that change the structure of the material over time [34]. Paper, usually produced from wood pulp, is subjected to hydrolysis, oxidation, and other chemical changes [35,36,37,38]. Hydrolysis, for example, involves the breakdown of cellulose into smaller molecules under the influence of water, which can cause the paper to lose its strength and flexibility [39]. The adhesive that holds the pages of a book together also undergoes similar changes. Adhesive bonds within the adhesive can weaken due to thermal, light, or moisture conditions, leading to a loss of elasticity. Oxidation of the adhesive can cause changes in color and texture, further affecting the binding strength and overall durability of the book.

The aim of this study was to investigate whether the initial elasticity and aging resistance of PVAc adhesive can be preserved or enhanced following the modification with SiO_2_ or TiO_2_ nanoparticles, given the previously established performance improvements associated with these modifications [30,31,32,33]. Along with the main aim of this study, two null hypotheses were formulated:

**H_01_:**  *There is no statistically significant difference between the material toughness of neat PVAc, nano-SiO_2_ PVAc, and nano-TiO_2_ PVAc adhesive.*

**H_02_:**  *There are no statistically significant changes in the FTIR spectra of paper–adhesive samples before and after exposure to UV irradiance for 48 or 96 h.*

## 2. Materials and Methods

### 2.1. Paper

For the purposes of the research, five different paper substrates were used. The basic properties of all used papers, according to the technical specifications of the manufacturers, along with corresponding abbreviations for the needs of this research, are presented in Table 1.

### 2.2. Nanoparticles

Odorless, solid, white powders of silica (SiO_2_) (Aerosil R 8200, Evonik Industries, Essen, Germany) and titanium dioxide (TiO_2_) (Aeroxide P25, Evonik Industries, Essen, Germany) nanoparticles were used for PVAc adhesive modifications. Both nanoparticles have approximately the same temped density (140 g/L) but a different BET surface area (135–185 m^2^/g SiO_2_; 35–65 m^2^/g TiO_2_) and assay based on ignited material (≥99.8% SiO_2_; ≥99.5% TiO_2_) [40,41].

### 2.3. Adhesives

Polyvinyl acetate (Signokol L, Signoplast, Zagreb, Croatia) adhesive is a water dispersion of vinyl acetate homopolymers with polyvinyl alcohol and the addition of plasticizers. It is mainly used as an adhesive for paper and board. The adhesive is supplied ready for use, and before application, it just needs to be stirred. In its liquid state, Signokol L is a white adhesive with a transparent dry film color. According to the material safety data sheet, its density is 1.0776 g/cm^3^ (20 °C), its viscosity is 8–10 Pa s (20 °C), its pH value is 6 ± 0.5, and its solid content is 45 ± 2% [42].

The modification of Signokol L PVAc adhesive was achieved by incorporating 1% silica (SiO_2_) or 1% titanium dioxide (TiO_2_) nanoparticles. To achieve the full potential of the nanocomposite, effective dispersion of nanoparticles in PVAc adhesive was achieved by using the digital IKA T 25 Ultra-Turrax homogenizer (IKA-Werke, Staufen, Germany) for 15 min. In the first 5 min, the mixing speed was continuously increased to 7000 revolutions per minute, and then it was maintained until the end of the mixing process. All prepared nano-modified PVAc adhesives were prepared using the same procedure under identical conditions (23 ± 1 °C; 50 ± 2% RH).

### 2.4. Testing of PVAc Adhesive’s Elasticity

#### 2.4.1. Tensile Properties and Test Procedures

**Figure 3:**
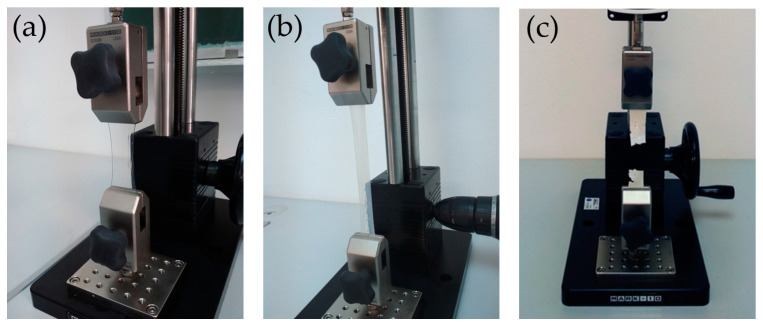
Tensile test procedure: (**a**) neat PVAc; (**b**) nano-SiO_2_ PVAc; (**c**) nano-TiO_2_ PVAc.

The determination of tensile properties was investigated according to the ISO 527-3:2018 standard [43]. Tensile properties were evaluated by placing a test specimen between two grips in the tensile MARK 10 ES30 (Mark-10, Copiague, NY, USA) testing machine (Figure 3). The tests were conducted under a relative humidity of 45 ± 2% and 23 ± 1 °C.

#### 2.4.2. Sample Preparation for Tensile Testing

**Figure 4:**
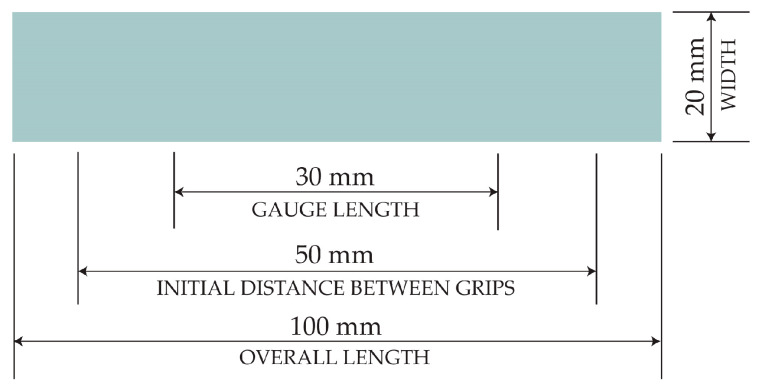
Dimension of the tensile test adhesive sample.

The form of the test samples was a 20 mm wide and 100 mm long strip with two parallel gauge marks, 30 mm apart, on the central portion of the sample. For each of the three adhesives (PVAc, nano-SiO_2_ PVAc, and nano-TiO_2_ PVAc), five stripes were selected from a total of twelve prepared samples. For samples preparation, silicon molds (100 × 25 × 2 mm) were used. In each mold, 5 mL of adhesive was placed and left to dry completely for 7 days at a relative humidity of 45 ± 2% and 23 ± 1 °C. To ensure that the 5 selected samples had no visible defects and had smooth edges, all samples were cut to 20 × 100 mm dimensions using a scalpel, and their thickness was measured with a micrometer. The final dimensions of the selected samples are illustrated in Figure 4. The number and size of samples for tensile testing were prepared according to the ISO 527-3:2018 standard [43].

### 2.5. Testing of PVAc Aging Resistance

#### 2.5.1. Sample Preparation for Adhesive Aging Resistance Testing

The preparation of samples for the surface characterization of materials, specifically FTIR spectroscopy, began with applying adhesives with a brush onto pre-cut sheets of paper measuring 210 × 99 mm. After drying completely for 48 h, the samples were cut into smaller dimensions necessary for UV radiation exposure and subsequently FTIR spectroscopy. The fabrication and drying of all samples occurred under conditions typical for graphic finishing and production processes, specifically at room temperatures between 18 °C and 20 °C and a relative humidity of 60% to 70%. Since it was essential to test three types of adhesive (PVAc, nano-SiO_2_ PVAc, and nano-TiO_2_ PVAc) in combination with five different types of paper (WFU, WFC, CW, WF_office_, and CR_office_), before exposure to UV radiation (0 h) and after 48 h and 96 h of UV radiation, a total of 45 samples measuring 40 × 20 mm had to be selected for research purposes. The size of the samples for testing the adhesive’s aging resistance was determined to be 40 × 20 mm so that all the samples required for testing a single UV radiation exposure time could fit simultaneously into the chamber. All adhesive tests were preceded by a seven-day conditioning of the samples under standard conditions according to ISO 187:1990 standard [44], 23 ± 1 °C and 50 ± 2% RH.

#### 2.5.2. FTIR Spectroscopy

The samples were characterized by Fourier-transform infrared spectroscopy with attenuated total reflection (ATR) using the Shimadzu IRAffinity-21 spectrometer (Shimadzu, Kyoto, Japan). The recordings were made in the spectral range of 4500 to 500 cm^−1^ with a resolution of 4 cm^−1^. The refractive index of the Specac Silver Gate Evolution ZnSe crystal is 2.4.

#### 2.5.3. Adhesive’s Resistance to UV Radiation

The adhesive’s resistance to UV radiation was tested by exposing samples in the Solarbox 1500e chamber (CO.FO.ME.GRA., Milano, Italy), according to the ISO 9142:2003 standard [45], for durations of 48 and 96 h under controlled conditions (550 W/m^2^ and 60 °C).

### 2.6. Statistical Analysis

In order to test the validity of the null hypotheses defined in the introduction part of this manuscript, analysis of variance (ANOVA) was used. To obtain the ANOVA *p*-values, based on which the null hypotheses can be rejected or accepted, Microsoft Excel software (Microsoft 365, version 2410) was used. The alpha significance level was, as usual, set to 0.05. One-way analysis of variance (ANOVA) was used to compare the means of material toughness of neat PVAc and nano-modified PVAc adhesives to determine if there is a statistically significant difference among them. The Bonferroni post hoc test was used after ANOVA to identify which specific pairs of groups are responsible for that difference. In addition, ANOVA was also used to compare the values of absorption peak intensities of neat PVAc and nano-modified PVAc adhesives before and after exposure to UV irradiance.

## 3. Results

### 3.1. PVAc Adhesive’s Elasticity

During the procedure, samples were exposed to continuous tension until breaking. For the purposes of this research, the elongation lengths $\left(l\right)$ of the adhesive sample strips were recorded at forces of 80 N, 90 N, 100 N, 110 N, 120 N, and 130 N. In order to generate a stress–strain curve, it is necessary to calculate the stresses (Equation (1)) and strains (Equation (2)) for each recorded force:

(1) $$\sigma =\frac{F}{S}$$

where σ is stress (N/mm^2^), F is applied force (N), and S is the cross-sectional area of a tested sample (mm^2^);

(2) $$\varepsilon =\frac{∆l}{l_{0}}$$

where ε is strain (unitless), ∆l is the change in the sample length after applied force (mm), and $l_{0}$ is the initial length of the part of the sample between the grips (mm).

The calculated tensile stresses of PVAc, nano-SiO_2_ PVAc, and nano-TiO_2_ PVAc adhesive test samples are listed in Table 2. Table 3 shows calculated tensile strains for tested adhesives.

**Figure 5:**
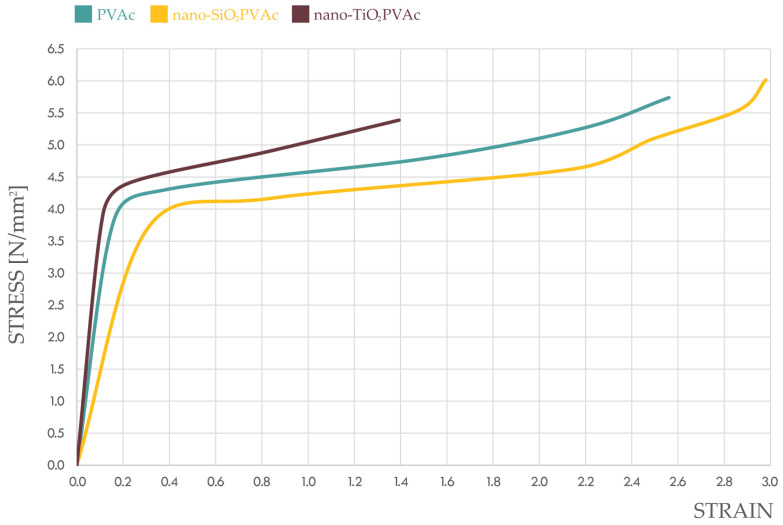
Tensile stress–strain curves of the tested adhesives (PVAc, nano-SiO_2_ PVAc, and nano-TiO_2_ PVAc).

Stress–strain curves were generated (Figure 5) according to the results listed in Table 2 and Table 3.

After creating the stress–strain curve, the area under the curve can be calculated. The area under the stress–strain curve $\left(U_{T}\right)\;$is an approximate value of the material toughness (Equation (3)). The larger the surface, the more energy will be needed to deform the material, i.e., the material has greater elasticity [46].

(3) $$U_{T}=\int_{0}^{\varepsilon_{f}}\sigma d\varepsilon$$

where $U_{T}$ is the toughness of the material (MPa), σ is the stress, ε is the strain, and $\varepsilon_{f}$ is the failure strain.

For a simpler comparison of the elasticity of the tested adhesives, it is necessary to calculate the adhesive stress and strain at the failure point and Young’s modulus of elasticity $\left(Ε\right)\;$according to Equation (4) [47]:

(4) $$Ε=\frac{\sigma_{f}}{\varepsilon_{f}}$$

where Ε is Young’s modulus of elasticity (GPa), $\sigma_{f}$ the failure stress (MPa), and $\varepsilon_{f}$ the failure strain (%). The calculated Young’s moduli of elasticity, the failure stresses, the failure strains, and the material toughness of PVAc, nano-SiO_2_ PVAc, and nano-TiO_2_ PVAc adhesives are listed in Table 4.

In Table 5, the one-way ANOVA results of the material toughness of adhesives are listed. The total number of groups was three (neat PVAc, nano-SiO_2_ PVAc, and nano-TiO_2_ PVAc), and five samples were tested for each adhesive. The source of variation of the material toughness had been divided into two categories, namely, between groups (BG) and within groups (WG). The F_statistic_ is the ratio of the mean square of BG to the mean square of WG. The probability of rejecting the first null hypothesis (H_01_) when it is true, i.e., the alpha significance level, was 0.05.

Bonferroni post hoc correction was performed in order to see which groups are different from the others. Results were obtained by performing individual *t*-tests and correcting the multiple comparisons with the Bonferroni correction method. The results are listed in Table 6. For the post hoc test, the alpha level needed to be adjusted via the Bonferroni method to account for the multiple hypotheses. The original (0.05) alpha level was divided by a number of intended comparisons (3). The Bonferroni corrected alpha level was 0.0167.

### 3.2. PVAc Adhesive’s Aging Resistance

**Figure 6:**
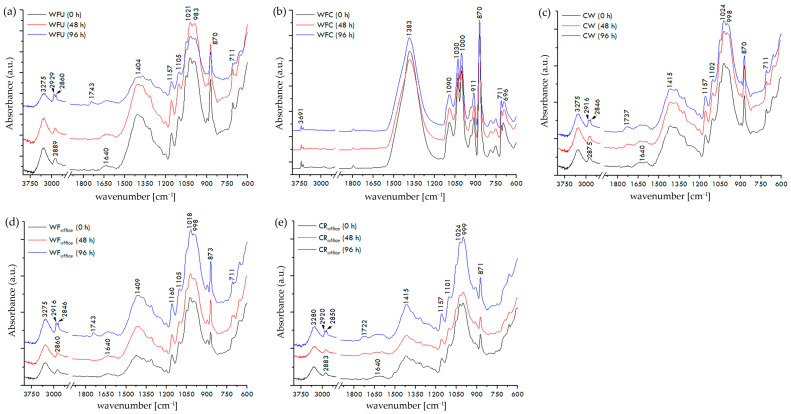
FTIR spectra of used paper samples before and after exposure to UV irradiance: (**a**) WFU, (**b**) WFC, (**c**) CW, (**d**) WF_office_, and (**e**) CR_office_.

FTIR spectra of paper samples (WFU, WFC, CW, WF_office_, and CR_office_) before and after exposure to UV radiation for 48 and 96 h are shown in Figure 6a–e. In the FTIR spectra of all uncoated papers (WFU, CW, WF_office_, and CR_office_), characteristic cellulose vibration bands (around 1415 cm^−1^, 1160 cm^−1^, and 983 cm^−1^), calcium carbonate bands (around 870 cm^−1^ and/or 711 cm^−1^), and adsorbed water bands (1640 cm^−1^) are visible [48,49,50]. The broad band around 3275 cm^−1^ in the spectra of these papers corresponds to the stretching of cellulose OH groups. Vibration bands around 2880 cm^−1^ likely indicate additives in the paper [51]. In the FTIR spectra of coated paper (WFC), characteristic coating bands composed of kaolin and calcium carbonate are visible. The broad band around 1383 cm^−1^, as well as bands at 3691 cm^−1^, 1090 cm^−1^, 1030 cm^−1^, 1000 cm^−1^, 911 cm^−1^, and 696 cm^−1^, indicate the presence of kaolin in the coating formulation. Vibration bands at 870 cm^−1^ and 711 cm^−1^ can be attributed to the vibration of CO_3_ bending [52].

During the aging process of paper, i.e., exposure to UV radiation, the formation of oxidative degradation products results in the formation of a broad band in the spectral range from 1550 to 1700 cm^−1^, where carbonyl groups appear. Carbonyl groups will only appear if the bending vibrations of adsorbed water molecules (1640 cm^−1^) are absent, as they can mask the products of cellulose oxidation [53]. Since adsorbed water molecules are present in all types of uncoated papers (WFU, CW, WF_office_, and CR_office_), the vibration bands of paper degradation products are probably masked.

Absorption bands of carboxyl or aldehyde groups of cellulose, resulting from opening of terminal glucopyranose rings or oxidation of C-OH groups, may appear above 1700 cm^−1^ [54]. After exposure of uncoated papers to UV radiation for 96 h, such absorption bands appear at 1743 cm^−1^ (WFU and WF_office_), 1737 cm^−1^ (CW), and 1722 cm^−1^ (CR_office_). The formation of -COOH may indicate the final oxidation state of carbon atoms in cellulose glucopyranose rings [55]. During UV radiation on uncoated papers, the vibration band around 2880 cm^−1^ splits into two, around 2920 cm^−1^ and 2850 cm^−1^, likely due to changes in C-H chains. Additional changes in the IR spectrum of the investigated papers during UV radiation are not observed. Coated (WFC) paper shows minimal or almost no changes, indicating the stability of the kaolin coating during aging (Figure 6b).

**Figure 7:**
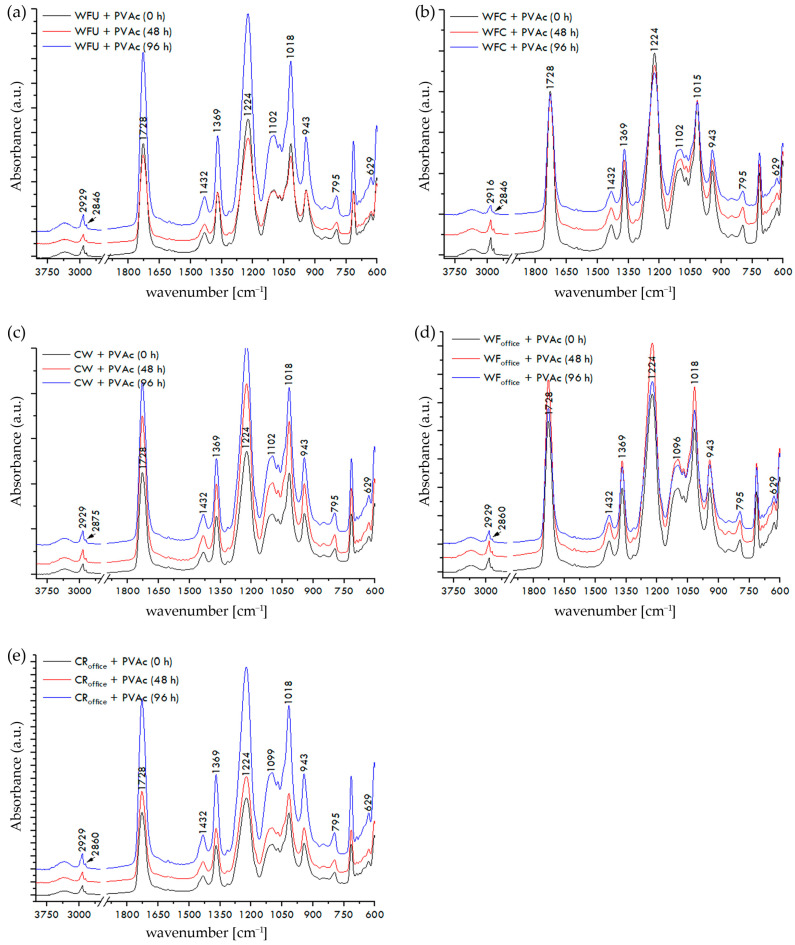
FTIR spectra of paper–PVAc adhesive samples before and after exposure to UV irradiance: (**a**) WFU + PVAc, (**b**) WFC + PVAc, (**c**) CW + PVAc, (**d**) WF_office_ + PVAc, and (**e**) CR_office_ + PVAc.

**Figure 8:**
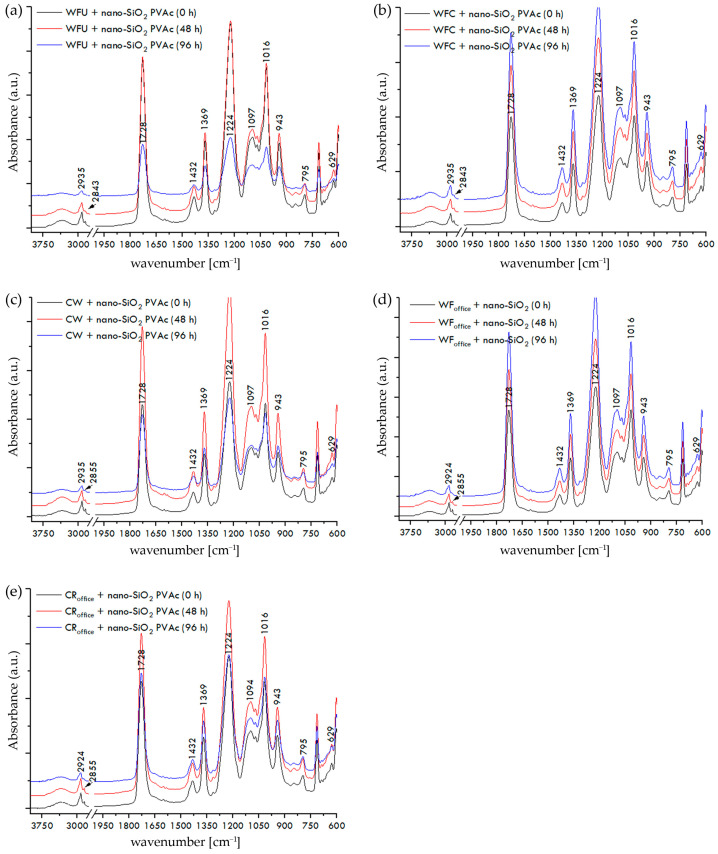
FTIR spectra of paper–nano-SiO_2_ PVAc adhesive samples before and after exposure to UV irradiance: (**a**) WFU + nano-SiO_2_ PVAc, (**b**) WFC + nano-SiO_2_ PVAc, (**c**) CW + nano-SiO_2_ PVAc, (**d**) WF_office_ + nano-SiO_2_ PVAc, and (**e**) CR_office_ + nano-SiO_2_ PVAc.

**Figure 9:**
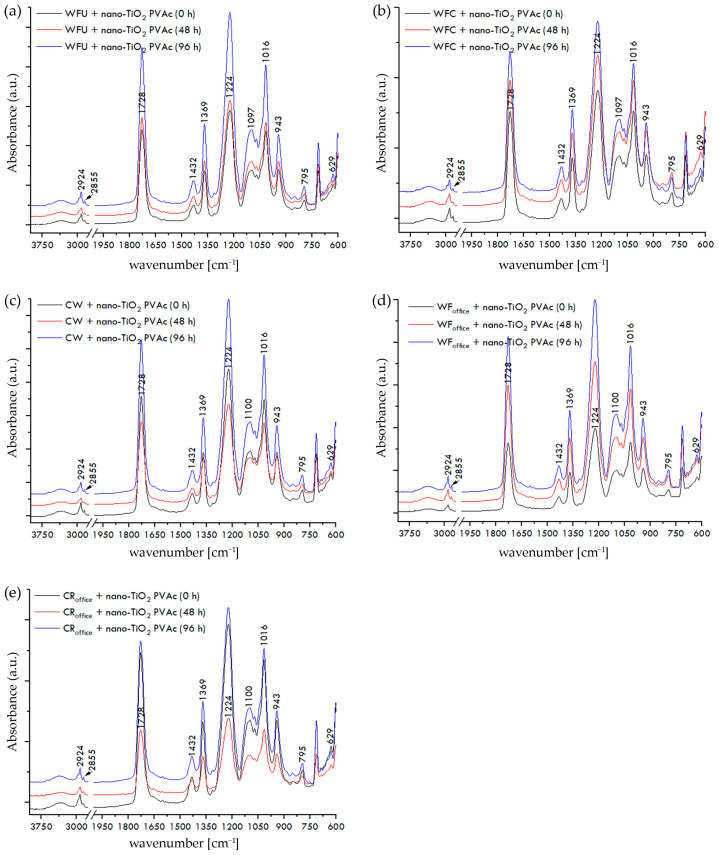
FTIR spectra of paper–nano-TiO_2_ PVAc adhesive samples before and after exposure to UV irradiance: (**a**) WFU + nano-TiO_2_ PVAc, (**b**) WFC + nano-TiO_2_ PVAc, (**c**) CW + nano-TiO_2_ PVAc, (**d**) WF_office_ + nano-TiO_2_ PVAc, and (**e**) CR_office_ + nano-TiO_2_ PVAc.

The FTIR spectra of paper–adhesive samples, recorded on the surface of the dried film of PVAc or nano-modified PVAc adhesive, before and after exposure to UV radiation for 48 and 96 h, are shown in Figure 7a–e, Figure 8a–e, and Figure 9a–e.

The paper is fully covered with adhesive, and the recorded spectra display the IR spectrum of PVAc. Vibrational bands in the range of 2935 to 2843 cm^−1^ correspond to the stretching vibrations of the CH, CH_2_, and CH_3_ groups characteristic of PVAc adhesives [56]. The stretching vibration of the carbonyl group of ester (C=O) acetate molecules at 1728 cm^−1^ is complemented by two less intense bands at 1432 cm^−1^ and 1369 cm^−1^, corresponding to the asymmetric and symmetric bending vibrations of CH_3_ groups. The vibrational band at 1224 cm^−1^ corresponds to the asymmetric stretching mode of the C-C(=O)-O ester group of PVAc, followed by bands at around 1097 cm^−1^ and 1016 cm^−1^, as well as a less intense band at 943 cm^−1^. Additionally, less intense peaks of C-H rocking vibrations are visible at 795 cm^−1^ and 629 cm^−1^ [57,58].

In Table 7, the one-way ANOVA *p*-value results of absorption peak intensities of paper–adhesive samples before and after exposure to UV irradiance are listed. The total number of groups was three (0 h, 48 h and 96 h), and 11 absorption peak intensities were tested for each group. The number of paper–adhesive samples was 15 (neat PVAc, nano-SiO_2_ PVAc, and nano-TiO_2_ PVAc with WFU, WFC, CW, WF_office_, and CR_office_ paper). The probability of rejecting the second null hypothesis (H_02_) when it is true, i.e., the alpha significance level, was 0.05.

## 4. Discussion

The adhesion bonding process is possible primarily due to the adhesion and cohesion attraction forces between the adhesive and the adherent. When joining two materials, PVAc adhesives form an adhesive film between them. The cohesive strength of this film influences the joint’s total strength. With adequate adhesion between the materials and high cohesive strength, the joint strength of the end-product will be exceptional. In our previous studies, through the modification of PVAc adhesive using SiO_2_ and TiO_2_ nanoparticles, we observed a rise in the cohesion strength of the adhesive, resulting in enhanced strength of the end-products [30,31]. This alteration theoretically led to a decrease in the adhesive’s elasticity [59].

Understanding the mechanical behavior of PVAc and nano-modified PVAc adhesives, particularly in relation to Young’s modulus of elasticity and stress–strain characteristics, is essential for optimizing their performance in different graphic arts applications.

The elasticity of the adhesive is greater when the Young’s modulus of elasticity $\left(Ε\right)\;$is lower, but the failure stress $\left(\sigma_{f}\right)\;$and the failure strain $\left(\varepsilon_{f}\right)\;$are higher. Based on the results presented in Table 4, we can conclude that the nano-SiO_2_ PVAc adhesive achieved the best results. The original PVAc adhesive follows, while the nano-TiO_2_ PVAc adhesive is considerably behind them. In addition, from the areas under the characteristic stress–strain curves of tested adhesives (Figure 5), which represent the toughness of the material $\left(U_{T}\right)$, it is evident that the nano-SiO_2_ PVAc adhesive has greater elasticity compared to the original PVAc adhesive and especially compared to the nano-TiO_2_ PVAc adhesive. A larger area under the stress–strain curve signifies higher material toughness. The adhesive with greater toughness has greater elasticity and undergoes a higher average stress compared to a brittle adhesive of equivalent strength. Before calculating the area under the curve, to numerically compare the changes in PVAc elasticity after nanoparticle modification, a one-way ANOVA test was performed in order to reject or accept the first null hypothesis (H_01_). According to Table 5, the ratio of the mean square of BG to mean square of WG, F_statistic_, is 269.501, much greater than F_critical_ (3.885). This means that the test was significant. In addition, the *p*-value was 1.067 × 10^−13^, much less than alpha significance for this test (0.05). The first null hypothesis (H_01_) is rejected, and the alternative hypothesis can be accepted: there is a statistically significant difference between the means of neat PVAc, nano-SiO_2_ PVAc, and nano-TiO_2_ PVAc adhesive. Results of Bonferroni post hoc correction (Table 6) showed that all adhesives’ groups were different from each other, because all *p*-values were less than the alpha significance level for this test (0.0167). This post hoc analysis revealed that the material toughness of nano-SiO_2_ PVAc adhesive (12.740 ± 0.311 MPa) was significantly larger compared with the neat PVAc adhesive (11.672 ± 0.239 MPa) and especially nano-TiO_2_ PVAc adhesive (6.481 ± 0.074 MPa). The elasticity of the adhesive, based on the material toughness, was increased by 9.15% by adding SiO_2_ nanoparticles. TiO_2_ nanoparticles significantly reduced the elasticity of PVAc adhesive by 44.47%.

The aging of papers and adhesives in books is a dynamic process that scientists are studying to preserve precious books and the heritage of words for future generations. Infrared spectroscopy enables deeper insight into degradation processes and the chemical changes in paper and adhesive that can occur during aging. Infrared spectroscopy with Fourier transformation (FTIR) is used in the determination of chemical bonds and functional groups in the formulation of paper and paper–adhesive samples, monitoring of polymerization reactions, and degradation reactions of tested samples [31,60,61].

FTIR spectra of all tested paper samples showed expected and characteristic bands, cellulose, calcium carbonate, and water bands for all uncoated paper samples, while bands indicating the presence of calcium carbonate and kaolin are shown on the FTIR spectrum of the coated paper samples. After UV radiation exposure for 48 and 96 h, coated paper samples showed minimal or almost no changes because of the stability of the kaolin coating. Unlike the uncoated paper samples, the coated paper samples exhibited exceptional stability and resistance to aging after UV radiation exposure for 48 and 96 h. Due to the presence of kaolin in their coating, only minimal or negligible changes were observed.

According to FTIR spectroscopy of paper–adhesive samples with neat PVAc and nano-modified PVAc adhesives, before their exposure to UV radiation, the addition of nanoparticles to the PVAc adhesive did not result in any significant changes in the IR spectra. After exposing all tested paper–adhesive samples to temperature changes, humidity fluctuations, and accelerated aging for 48 and 96 h, significant changes in the IR spectra were not observed either, indicating the relative stability of the PVAc and nano-modified PVAc adhesive films on the surface of the paper against UV radiation for the selected exposure times. Although no significant changes were observed in the FTIR spectra of paper–adhesive samples, an ANOVA test was conducted to confidently accept the second null hypothesis (H_02_). All obtained *p*-values were in range of 0.0645–0.9378, which means that *p*-values for all investigated paper–adhesive samples were greater than the alpha significance level for this test (0.05). Therefore, the second null hypothesis (H_02_) is accepted: there are no statistically significant changes in the FTIR spectra of paper–adhesive samples before and after exposure to UV irradiance for 48 or 96 h.

While it was anticipated that this study will assist engineers and designers in selecting suitable adhesives and nanoparticles, it is important to highlight certain methodological limitations. Addressing these limitations is essential for achieving a deeper and more comprehensive understanding of the potential use of nanoparticles in modifying PVAc adhesives in future research. While SiO_2_ and TiO_2_ nanoparticles are commonly utilized and provide valuable insights into PVAc adhesive modification, future research could explore a broader range of nanoparticles to achieve enhanced or novel properties. In this study, paper–adhesive samples were exposed to UV irradiation for 48 and 96 h. However, the duration of exposure may be insufficient to fully understand the long-term aging behavior of nano-modified PVAc adhesives. Additionally, this study is primarily focused on the elastic properties of neat and nano-modified PVAc adhesives, but it did not investigate their elastic performance in specific practical applications. Future research could expand testing to include application-specific performance evaluations to better assess the real-world effectiveness of these adhesives. Moreover, future studies should delve deeper into the fundamental properties of adherent materials selected for graphic applications and examine their interactions with both neat and nano-modified PVAc adhesives. For instance, when using uncoated paper in book production, sufficient elastic adhesives can prevent internal tearing of the paper, enabling books to lie flat when open, while maintaining strong binding and providing excellent user experience. In contrast, coated papers may present challenges as the adhesive forces between the coating and the paper diminish. While the coated layer may initially adhere to the adhesive, prolonged use could lead to disintegration of the binding, regardless of the adhesive’s elasticity or the aging resistance of the paper and adhesive. The discovery that SiO_2_ nanoparticles improve the flexibility of PVAc adhesives opens the door to further research into enhancing durability and flexibility for a broader range of applications. As sustainability becomes a priority, future studies should investigate how nanoparticles affect the environmental footprint of PVAc adhesives, including biodegradability and toxicity. Ultimately, these findings could pave the way for next-generation PVAc adhesives that are more efficient, durable, and eco-friendly, expanding their usability across various industries.

## 5. Conclusions

Polyvinyl acetate (PVAc) adhesives become more elastic after modification with silica (SiO_2_) nanoparticles, allowing the joint to move more freely without sacrificing integrity. Instead of concentrating force at specific points, stress is distributed over a larger area, effectively reducing the risk of failure and enhancing the overall strength of the bond. In addition, the more elastic adhesive also offers better end-user experience; for example, it allows flat book-opening behavior. The modification of the PVAc adhesive with titanium dioxide (TiO_2_) nanoparticles did not yield the desired outcome in terms of increasing or preserving elasticity. Unfortunately, this modification led to decreased elasticity compared to the neat PVAc adhesive; therefore, nano-TiO_2_ PVAc adhesive could be classified as brittle. Bittle adhesives are more sensitive to crack propagation, have lower fatigue life, and may not be suitable for applications requiring flexibility, such as in the production of reusable graphic products, for example, books. The modification of PVAc adhesives with SiO_2_ or TiO_2_ nanoparticles did not lead to any significant changes in their IR spectra. Since PVAc adhesives generally show satisfactory resistance to aging, it is established that this property remains unchanged by the addition of nanoparticles, as all adhesives exhibit nearly identical behavior after exposure to UV radiation for 48 and 96 h. Therefore, by incorporating SiO_2_ or TiO_2_ nanoparticles, it is possible to preserve the desirable property of PVAc adhesive: satisfactory stability of the PVAc film under aging effects. This insight emphasizes that while nanoparticles can enhance certain properties of adhesives, careful consideration of their effects on other properties is crucial to ensure suitability for specific applications.

## Figures and Tables

**Table 1:** Properties and abbreviations of paper samples used.

Paper Sample	Woodfree Uncoated	Woodfree Coated	Bulky (Containing Wood)	Office Paper (Virgin Fibers)	Office Paper (Recycled Fibers)
Abbreviation	WFU	WFC	CW	WF_office_	CR_office_
Grammage [g/m^2^]	100	115	90	80	80
Roughness (Bendtsen) [mL/min]	160	80	300	120	225

**Table 2:** Calculated tensile stresses $\left(\sigma \right)$ of PVAc, nano-SiO_2_ PVAc, and nano-TiO_2_ PVAc adhesive test samples.

F[N]	σ [N/mm^2^]		
	PVAc (Mean ± SD)	Nano-SiO_2_ PVAc (Mean ± SD)	Nano-TiO_2_ PVAc (Mean ± SD)
80	3.826 ± 0.050	3.701 ± 0.048	3.918 ± 0.018
90	4.304 ± 0.056	4.164 ± 0.054	4.408 ± 0.020
100	4.783 ± 0.062	4.627 ± 0.060	4.898 ± 0.022
110	5.261 ± 0.068	5.089 ± 0.066	5.388 ± 0.025
120	5.739 ± 0.075	5.552 ± 0.072	Break
130	Break	6.015 ± 0.078	Break

**Table 3:** Calculated tensile strains $\left(\varepsilon \right)$ of PVAc, nano-SiO_2_ PVAc, and nano-TiO_2_ PVAc adhesive test samples.

F[N]	ε [unitless]		
	PVAc (Mean ± SD)	Nano-SiO_2_ PVAc (Mean ± SD)	Nano-TiO_2_ PVAc (Mean ± SD)
80	0.160 ± 0.044	0.307 ± 0.044	0.113 ± 0.045
90	0.387 ± 0.062	0.833 ± 0.079	0.227 ± 0.049
100	1.493 ± 0.077	2.153 ± 0.088	0.827 ± 0.085
110	2.187 ± 0.045	2.487 ± 0.054	1.393 ± 0.065
120	2.560 ± 0.077	2.867 ± 0.076	Break
130	Break	2.980 ± 0.081	Break

**Table 4:** Young’s moduli of elasticity $\left(Ε\right)$, failure stresses $\left(\sigma_{f}\right)$, failure strains $\left(\varepsilon_{f}\right)$, and material toughness $\left(U_{T}\right)$ of PVAc, nano-SiO_2_ PVAc, and nano-TiO_2_ PVAc adhesives.

Parameter	PVAc (Mean ± SD)	Nano-SiO_2_ PVAc (Mean ± SD)	Nano-TiO_2_ PVAc (Mean ± SD)
Ε [GPa]	0.031 ± 0.011	0.015 ± 0.003	0.043 ± 0.020
$\sigma_{f}\;$[MPa]	5.739 ± 0.075	6.015 ± 0.078	5.420 ± 0.048
$\varepsilon_{f}$ [%]	256.0 ± 7.71	298.0 ± 8.04	139.3 ± 6.47
$U_{T}$ [MPa]	11.672 ± 0.239	12.740 ± 0.311	6.481 ± 0.074

**Table 5:** One-way ANOVA result of the material toughness $\left(U_{T}\right)$ of neat PVAc, nano-SiO_2_ PVAc, and nano-TiO_2_ PVAc adhesive for testing the validity of hypothesis H_01_.

Source of Variation	SS	df	MS	F_statistic_	*p*-Value	F_critical_
Between Groups	112.114	2	56.057	269.501	1.067 × 10^−13^	3.885
Within Groups	2.496	12	0.208			

Number of groups = 3; number of observations = 15; alpha level = 0.05.

**Table 6:** Bonferroni post hoc procedure for identifying the pairwise differences between the group means after ANOVA.

Groups	*p*-Value (*t* Test)	Significant?
PVAc v nano-SiO_2_ PVAc	0.0122	Yes
Nano-SiO_2_ PVAc v nano-TiO_2_ PVAc	1.577 × 10^−8^	Yes
Nano-TiO_2_ PVAc v PVAc	3.065 × 10^−8^	Yes

*t* test tails = 2; *t* test type = 2; alpha level = 0.0167.

**Table 7:** ANOVA *p*-value results of absorption peak intensities of paper–adhesive samples before and after exposure to UV irradiance for testing the validity of hypothesis H_02_.

Paper–Adhesive Sample	*p*-Value
WFU + PVAc	0.1934
WFC + PVAc	0.7013
CW + PVAc	0.4866
WF_office_ + PVAc	0.7815
CR_office_ + PVAc	0.0669
WFU + nano-SiO_2_ PVAc	0.0645
WFC + nano-SiO_2_ PVAc	0.5631
CW + nano-SiO_2_ PVAc	0.1177
WF_office_ + nano-SiO_2_ PVAc	0.5664
CR_office_ + nano-SiO_2_ PVAc	0.5123
WFU + nano-TiO_2_ PVAc	0.5222
WFC + nano-TiO_2_ PVAc	0.9378
CW + nano-TiO_2_ PVAc	0.4579
WF_office_ + nano-TiO_2_ PVAc	0.2361
CR_office_ + nano-TiO_2_ PVAc	0.1284

Number of groups = 3; number of observations = 33; alpha level = 0.05.

## Data Availability

The original contributions presented in the study are included in the article, further inquiries can be directed to the corresponding authors.
